# Torsion of the Accessory Spleen in a Child With Acute Abdomen

**DOI:** 10.7759/cureus.38643

**Published:** 2023-05-06

**Authors:** Mehmet Çakmak, Nazli Gulsum Akyel

**Affiliations:** 1 Pediatric Surgery, Başakşehir Çam and Sakura City Hospital, İstanbul, TUR; 2 Pediatric Radiology, Şanlıurfa Training and Research Hospital, Şanlıurfa, TUR

**Keywords:** atypical abdominal pain in children, abdominal pain in children, i̇ntra-abdominal mass, ultrasonography for abdominal pain, abdominal pain, acute abdomen, accessory splenic torsion, accessory spleen

## Abstract

Accessory splenic torsion is a rare condition that occurs when the accessory spleen twist on its pedicle, leading to a loss of blood supply and subsequent tissue damage. It is a rare cause of acute abdomen with few cases reported in the literature. We report a case of accessory spleen torsion in a 16-year-old male with abdominal pain. The patient, whose lesion was interpreted as a hematoma on imaging at an external center, was admitted to our center with increased, intermittent abdominal pain. The patient's complaints and physical examination were similar to peptic ulcer perforation. Abdominal ultrasonography and abdominal CT performed for differential diagnosis showed a 45x50 mm heterogeneous, hypodense, well-defined lesion located in the splenic hilus, posterior to the stomach, and adjacent to the pancreatic tail. In our center, the lesion was considered to be lesser sac omental torsion and was operated on. A 720-degree torsed accessory spleen was found at surgery and resected. Accessory splenic torsion is not primarily a condition that comes to mind in children with abdominal pain. However, in case of delay in diagnosis and treatment, many complications can be seen. The fact that ultrasonography or computed tomography cannot clearly define accessory splenic torsion also complicates this diagnosis. In such cases, performing diagnostic laparotomy/laparoscopy reveals the definitive diagnosis and is very important in preventing complications.

## Introduction

The accessory spleen is a congenital anomaly of the spleen, and its prevalence has been reported to be 10-30% of the population [1-3]. The spleen appears in several parts in the fifth week of embryogenesis and fuses shortly before birth. In the event of any deficiency in this fusion, an accessory spleen may be observed [4]. Accessory spleens are most common in the hilum of the spleen (75%) and the tail of the pancreas (20%) [5]. It is usually asymptomatic or incidentally detected on imaging or laparotomy performed for other reasons. Asymptomatic cases detected on imaging do not require surgical intervention. Rarely, accessory spleens may torsion and cause symptoms of acute abdomen. Untreated cases can cause hemorrhagic shock, peritonitis, or rupture [6]. It is not uncommon for accessory spleen torsion to be misdiagnosed on imaging as an abdominal mass, as the symptoms and imaging findings can be nonspecific. In these lesions, clinicians and radiologists should consider the diagnosis, and a biopsy should not be performed. The diagnosis can be challenging and is usually made through surgery. Treatment involves the resection of the torsed accessory spleen. Herein, we report a case of an accessory spleen torsion in a 16-year-old male with abdominal pain.

## Case presentation

**Figure 1:**
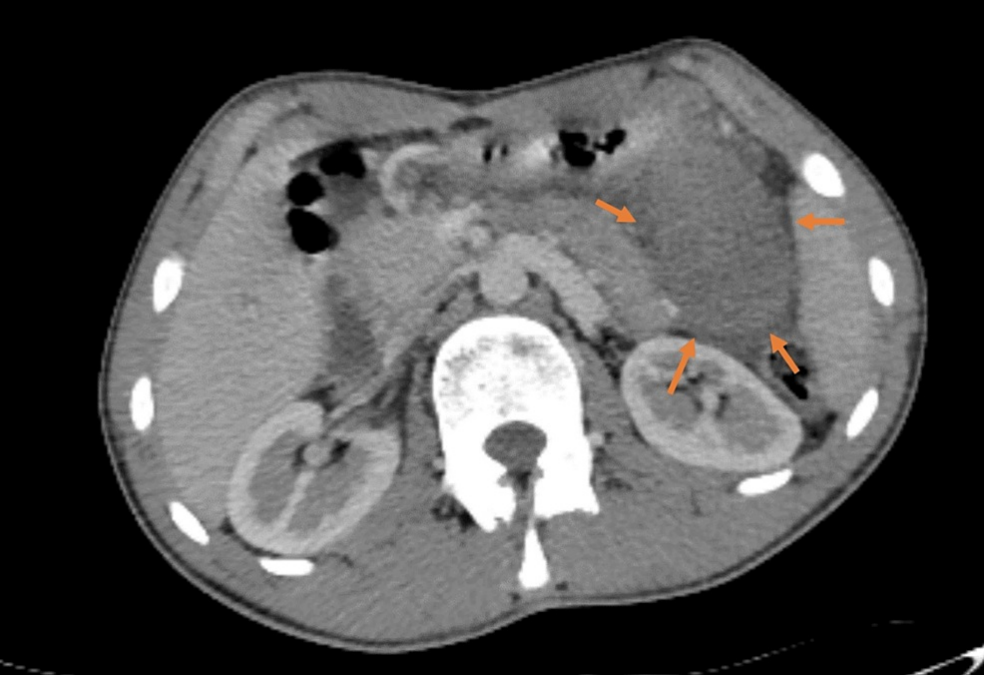
Axial contrast-enhanced CT image shows a well-defined hypodense mass compared to the adjacent spleen and pancreatic tail.

A 16-year-old male patient was presented to the emergency room with intermittent abdominal pain for the last month, without any history of trauma. Over the course of a month, he initially had tolerable pain, but his symptoms gradually increased over the last three days. He described cramping pain in the epigastric region that increased after eating. There was no change in pain during respiratory movements. He had nausea but no vomiting. His vitals were stable, and his fever was around 36.5-37 °C. Physical examination revealed tenderness from the epigastric region extending to the left upper quadrant of the abdomen. He had no defense. His sclera was icteric, but his skin color was normal. The patient’s complaints and physical examination were primarily suggestive of a peptic ulcer perforation. No significant findings were found in other laboratory tests, except for total bilirubin level 3.21 mg/dL (the reference range 0-1 mg/dL), indirect bilirubin level 2.96 mg/dL (the reference range 0-0,7 mg/dL), and C-reactive protein 127 mg/L (the reference range 0-5 mg/L) tests. Abdominal ultrasonography revealed a 45x46 mm mass containing cystic and heterogenous hyperechogenic areas with septations. The lesion was located in the splenic hilus adjacent to the tail of the pancreas, and there was no vascular coding on Doppler examination. An abdominal CT performed with intravenous contrast material for differential diagnosis showed a 45x50 mm heterogeneous, hypodense, well-defined lesion located in the splenic hilus, posterior to the stomach, and adjacent to the pancreatic tail (Figure 1).

**Figure 2:**
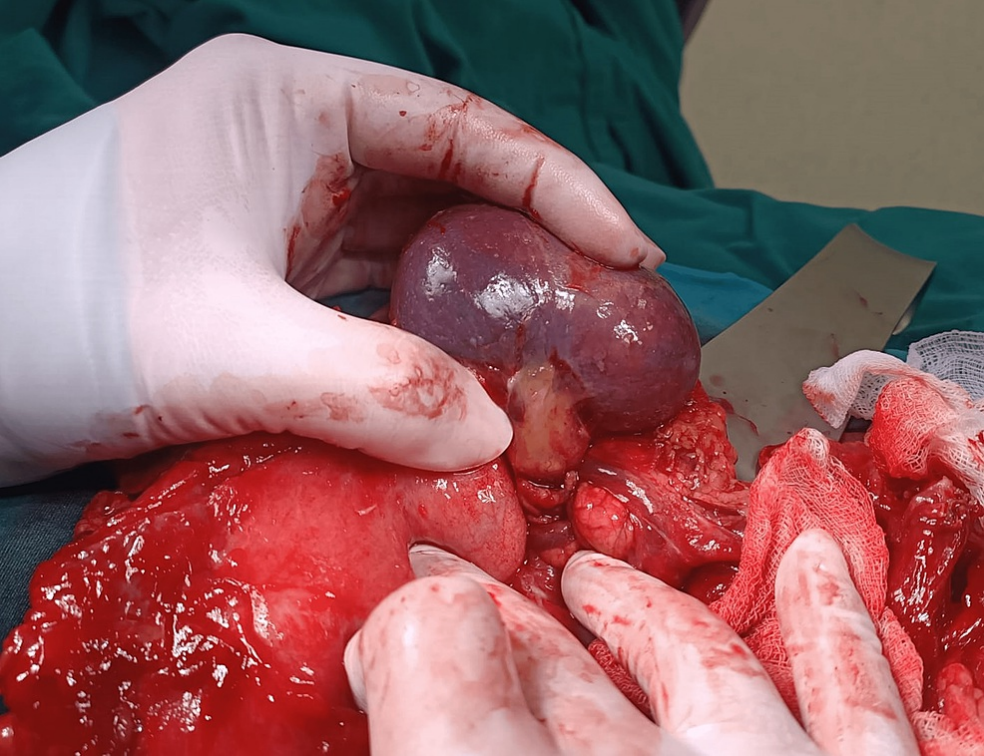
The intraoperative image of the accessory spleen was rotated at 720-degrees.

Lesser sac omental torsion was considered in the differential diagnosis. Since there was no history of trauma and the hemoglobin was normal, the diagnosis of a hematoma was ruled out. After 12 hours of clinical follow-up, abdominal pain and epigastric tenderness persisted, so a laparoscopy was performed for diagnosis and treatment. Laparoscopic observation revealed serous fluid in the gastrocolic region and rectovesical space. When the gastrocolic ligament was opened to access the mass, the posterior wall of the stomach and adjacent mesenteric tissues were highly inflamed. The laparoscopic procedure was terminated, and the abdomen was explored through a median incision above the umbilicus. No fistula or perforation was found in the posterior wall of the stomach. With blunt dissections, the accessory spleen, approximately 40x50 mm in size and twisted 720-degrees clockwise, was found adjacent to the pancreatic tail (Figure 2).

**Figure 3:**
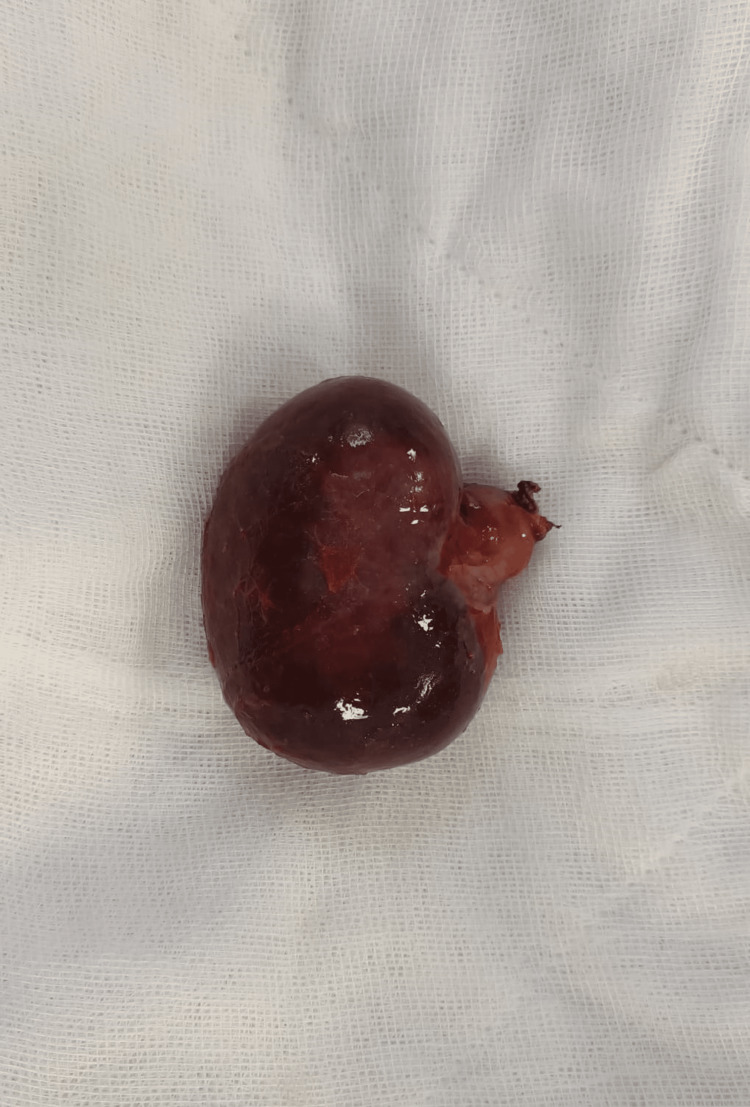
The image of a torsioned accessory spleen after excision.

The detorsion and subsequent excision of the accessory spleen were done (Figure 3). The pain regressed in the postoperative follow-up. In postoperative laboratory tests, total bilirubin and indirect bilirubin levels decreased and reached the reference range after 72 hours. The patient was discharged on the fourth postoperative day.

## Discussion

The accessory spleen can be diagnosed at any stage of life, from early childhood to old age. Surgical intervention is not required in asymptomatic patients, but accessory spleens, which are encountered incidentally in any abdominal surgery and may cause future complications, should be excised. Complaints of abdominal pain in children are often atypical for their age group, and physical examination is difficult due to patient non-compliance. Especially in patients with abdominal pain, restlessness, vomiting, and fever; ultrasonography may be the first choice if there is a suspicious finding on physical examination regarding the acute abdomen. Although there are many causes of acute abdomen pain, accessory splenic torsion, which is a rare cause, is often difficult to diagnose and can lead to infarction, acute inflammation, spontaneous rupture, hemorrhagic shock, intestinal obstruction, and peritonitis if the diagnosis is missed [6]. Hematoma, abscess, cyst, and tumor may be considered in the differential diagnosis. However, it is difficult to detect a twisted pedicle on ultrasound in accessory splenic torsion [7]. The lack of contrast enhancement on contrast-enhanced CT makes diagnosis difficult. In our case, ultrasonography revealed only a 45x46 mm mass and no evidence of accessory splenic torsion. In the abdominal tomography performed with an intravenous contrast material for differential diagnosis, a hypodense lesion with a smoothly circumscribed appearance was detected in the splenic hilus, posterior to the stomach, adjacent to the pancreatic tail. Ultrasonography and CT results were not suggestive of torsion of the accessory spleen, which made the diagnosis difficult. A diagnostic laparoscopy was performed due to the persistence of abdominal pain and epigastric tenderness in the 12-hour clinical follow-up of the patient, and the definitive diagnosis could only be reached through laparoscopy. Magnetic resonance imaging and scintigraphy can be used in the differential diagnosis, but may not be possible in all patients due to potential delays in clinical care [8].

## Conclusions

Accessory splenic torsion is rare and difficult to diagnose, especially in children. The failure to detect significant pedicle torsion on ultrasonography and CT may have led to the missed diagnosis of these cases. Since accessory spleens are most commonly seen in the anterior or medial splenic and pancreatic tail regions, accessory splenic torsion should be considered in children with abdominal pain if a mass is found accompanied by abdominal tenderness. If difficulty is encountered in diagnosis, a diagnostic laparoscopy/laparotomy should be performed. Otherwise, a torsioned accessory spleen may cause an infarction, hemorrhage, abscess, sepsis, mechanical compression of adjacent organs, and many other complications.
